# SMARCB1-deficient poorly differentiated testicular carcinoma: a case report

**DOI:** 10.3389/fonc.2025.1554352

**Published:** 2025-03-06

**Authors:** Zhiying Wang, Zhixian Zhong, Yi Zhong, Cunya Li, Yun Li, Ling Xu, Shujuan Fu

**Affiliations:** ^1^ Department of Oncology I, Yueyang Hospital of Integrated Traditional Chinese and Western Medicine, Shanghai University of Traditional Chinese Medicine, Shanghai, China; ^2^ Department of Oncology, East Hospital Affiliated to Tongji University, School of Medicine, Tongji University, Shanghai, China; ^3^ Oncology Department, Shanghai TCM-Integrated Hospital, Shanghai University of Traditional Chinese Medicine, Shanghai, China

**Keywords:** SWI/SNF, SMARCB1, INI1-deficient, rare diseases, testicular cancer

## Abstract

In the present study, a 36-year-old male presented with left scrotal enlargement without an obvious cause, accompanied by a feeling of heaviness. Imaging examinations revealed a left testicular malignancy, the patient underwent left testicular mass removal,and the postoperative pathology results revealed a highly malignant germ cell tumor, with a tendency toward poorly differentiated embryonal carcinoma or seminoma. After surgery, the condition of the patient deteriorated rapidly, and distant tumor metastasis occurred. Lymph node puncture pathology results revealed poorly differentiated carcinoma consistent with SMARCB1/INI-1 deletion. Despite the use of chemotherapy, radiotherapy, immunotherapy and targeted therapy, the patient died 11 months after surgery. To the best of our knowledge, this is the first case report of a SMARCB1/INI1-deficient Poorly differentiated testicular carcinoma, which is very similar to testicular spermatocytic carcinoma in clinical diagnosis and deserves differentiation for future clinical diagnoses.This report provides important insights into the diagnosis and treatment of SMARCB1/INI1-deficient testicular malignancy. SMARCB1 is a crucial tumor suppressor gene, and its deficiency is closely associated with the development of various malignant tumors. The identification of this case suggests that future research should further explore the molecular mechanisms of SMARCB1-deficient tumors, particularly their role in testicular malignancies. Additionally, the diagnostic process of this case highlights that SMARCB1/INI1-deficient tumors can be clinically very similar to spermatocytic carcinoma of the testis, which can easily lead to misdiagnosis. Therefore, future clinical practice should emphasize the detection of SMARCB1/INI1 expression status, especially in the context of highly aggressive and rapidly progressing testicular malignancies, where immunohistochemical testing for SMARCB1/INI1 should be considered to confirm the diagnosis. In terms of treatment, this case demonstrates the highly aggressive nature and resistance to conventional therapies of SMARCB1/INI1-deficient tumors. Despite the patient receiving multiple treatments, disease progression could not be halted. This underscores the need for the development of novel therapeutic strategies targeting SMARCB1/INI1-deficient tumors, such as combinations of immune checkpoint inhibitors and targeted therapies, or other emerging immunotherapeutic approaches. Moreover, the treatment course of this patient also reflects the importance of individualized treatment plans. Future research should further explore precision medicine strategies based on tumor genetic profiles to improve patient survival rates and quality of life.

## Introduction

Globally, testicular cancer is the most common malignant tumor in young men between the ages of 15 and 40, constituting 1% of male cancer cases and 5% of urological malignancies (1, 2). Despite the high incidence of testicular cancer, mortality rates have declined, primarily due to the introduction of platinum-based chemotherapy in the 1970s (3). Testicular cancer is highly curable, but its unsatisfactory life quality and adverse side effects remain major health problems (4). Not only the disease itself, but also the treatments, such as cisplatin-based combination chemotherapy and post-chemotherapy surgical procedures, affect semen quality and male reproductive function (5). All normal cells express switch/sucrose non-fermentable (SWI/SNF)-related matrix-associated actin-dependent regulator of chromatin subfamily B member 1 (SMARCB1) [also known as integrase interactor 1 (INI1)] as part of the SWI/SNF ATP-dependent chromatin remodeling complex (6). Aberrant expression of SMARCB1 has been found in a variety of tumors, such as pancreatic rhabdoid carcinoma (7), yolk sac tumors (8) thoracic neoplasms (9), soft-tissue neoplasms (10) and renal cancer (11). SMARCB1 is a tumor suppressor that can prevent rapid cell proliferation. As a core subunit of the SWI/SNF complex, it plays a role in ATP-dependent chromatin remodeling, thereby regulating gene expression, and is associated with a variety of cell functions, including DNA damage repair and cell growth regulation (12). SMARCB1/INI1-deficient cancer is highly invasive (13). There is currently a lack of reports on SMARCB1/INI1-deficient testicular cancer. The present study reports, to the best of our knowledge, the first case of a SMARCB1/INI1-deficient testicular malignancy, which is very similar to testicular spermatocytic carcinoma in clinical diagnosis and deserves differentiation for future clinical diagnosis.

## Case report

In July 2022, a 36-year-old male patient with no family history of malignant tumors developed left scrotal enlargement without any obvious cause, accompanied by a feeling of heaviness. The patient was admitted to the Shanghai Hospital of Traditional Chinese and Western Medicine for treatment. The scrotal ultrasound report indicated an enlargement of the left testicle, with a possible testicular lesion. Due to the simple structure of the testicles, the patient did not undergo a CT scan prior to surgery. After the exclusion of related contraindications, For example, distant transfers, and complete preoperative examinations, the patient underwent left testicular mass removal under general anesthesia in the Department of Urology, Renji Hospital (Shanghai, China) in August 2022. The postoperative pathology results revealed a highly malignant germ cell tumor of the testicle, with features similar to those of poorly differentiated embryonal carcinoma or seminoma. Given the tumor’s location in the testis and the patient’s relatively young age, the initial consideration is a germ cell-derived malignant tumor. Although the tumor exhibited negative expression of octamer-binding transcription factor 3/4 (OCT3/4), this type of tumor is not commonly associated with mutations in the INI1 gene, which explains why it was not reflected in the diagnosis. Therefore, after surgery, the patient was diagnosed with testicular spermatogonial carcinoma.

**Figure 1:**
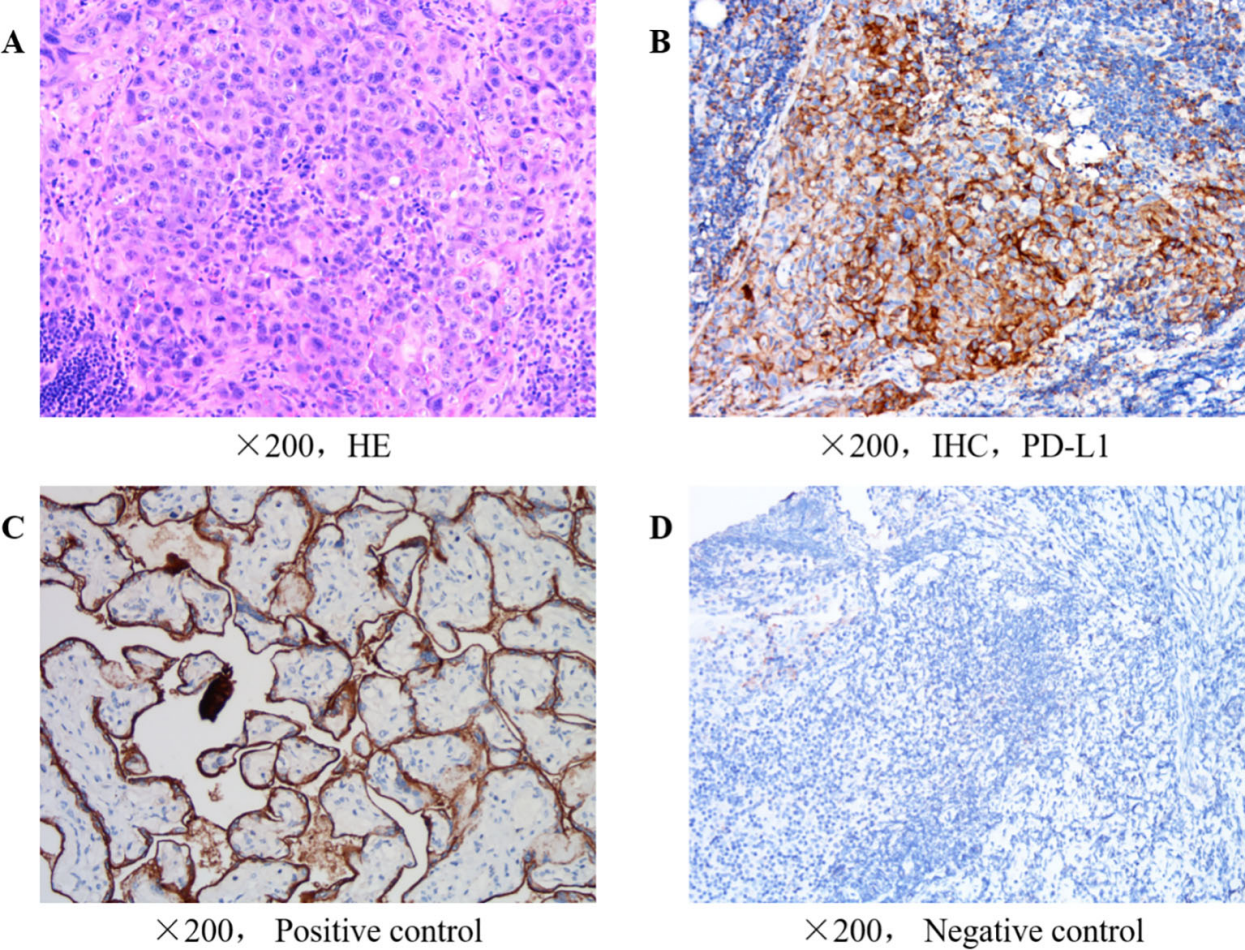
**(A)** H&E of lymph nodes in the neck. **(B)** Immunohistochemical of cervical lymph nodes for PD-L1 expression. **(C)** Positive control for immunohistochemical of cervical lymph nodes for PD-L1 expression. **(D)** Negative control for immunohistochemical of cervical lymph nodes for PD-L1 expression.

The immunohistochemistry of the postoperative tumor tissue showed that cytokeratin(+), placental alkaline phosphatase (partial -/+), vimentin(-), cytokeratin CAM5.2(+), sal-like protein 4 (foci +), Ki67(65% +), human chorionic gonadotropin(-), CD34(-), Wilms’ tumor 1(-), calretin(-), D2-40(-), CD30(-), OCT3/4(-), programmed death ligand 1 (PD-L1)[combined positive score (CPS=2)](CPS was calculated as the number of PD-L1 staining tumor and immune cells divided by the total viable tumor cells multiplied by 100 with a maximum score of 100. )14, INI-1(-), CD117(-), Brahma-related gene-1(+), CD56(-), chromogranin A(-), synaptophysin(-) and CD138(-). According to the pathology results and the diagnose of testicular spermatogonial carcinoma, from the first postoperative period to the fourth month after surgery, the patient received 4 courses of postoperative adjuvant chemotherapy, with the following regimen: Cisplatin (30 mg intravenously on days 1to day 5,every 3 weeks), etoposide (0.1 g, intravenously,on days 1-5,every 3 weeks) and bleomycin (15,000 units intramuscular injection on days 1, 3 and 5,every 3 weeks), which is first-line adjuvant chemotherapy regimen for postoperative testicular seminoma.The treatment went smoothly, and the patient reported no obvious adverse reactions to chemotherapy, such as bone marrow suppression. However, in the postoperative follow-up in December of the same year, the patient presented with swollen lymph nodes in the neck, and a positron emission tomography-CT scan performed at Xinhua Hospital (Shanghai, China) showed metastases in multiple bones in the body (lower sternum, left femur, left scapula, right 6th posterior rib, left 5th rib and bilateral iliac bones), multiple regional lymph nodes (left side of the neck, left clavicle, right retrophrenic angle and retroperitoneum) and the right upper lobe of cerebrum. Multiple lymph nodes in both sides of the neck showed low or slightly high metabolic activity, which was more prominent on the left side, indicating a high risk of metastasis. The immunohistochemistry analysis of a lymph node tissue biopsy was performed in Shanghai Integrated Traditional Chinese and Western Medicine Hospital Affiliated to Shanghai University of Traditional Chinese Medicine January 2023,the result revealed the following: PD-L1 tumor proportion score (indicating protein level), 30%; combined proportion score 40% (**Figure 1**); the immune cell score 10%; tumor mutational burden, 2.79/Mb; and microsatellite stability 14. Potential effective chemotherapies consisted of oxaliplatin, carboplatin, cisplatin, vincristine, and the chemotherapy normally used for this tumor type, gemcitabine.

Although the patient’s tumor PD-L1 expression and lymph node puncture PD-L1 expression were both not high at the time of surgery, referring to the relevant literature on SMARCB1-deficient tumor immunotherapy, which has shown the potential of immunotherapy for the treatment of SMARCB1-deficient tumors, we believe that the patient may still benefit from immunotherapy. Another important reason we chose to further use immunotherapy is that the patient’s immune microenvironment changes with treatment. Although the pathology after surgery indicated a CPS of 2, the patient’s lymph node PD-L1 expression after treatment showed a trend of tumor sensitivity to immunotherapy. Therefore, we believe that the patient may still benefit from immunotherapy. Ultimately, we selected cadonilimabb, a dual-antibody immunotherapy agent. Previous studies have shown that regardless of whether PD-L1 expression is positive or negative, the objective response rate (ORR) is 33.0%, with a complete response (CR) rate of 12.0%.

From January to March 2023, the patient underwent three cycles of chemotherapy combined with immunotherapy, specifically: 375 mg of cadonilimab intravenously on day 1 every 3 weeks, plus 300 mg of cisplatin intravenously (ivgtt) on day 1 and day 8, every 3 weeks. Starting from February, the patient began oral targeted therapy with anlotinib (8 mg orally on days 1-14, every 3 weeks). 21 days after the end of the last chemotherapy session the patient developed intracranial hypertension and was treated with mannitol and glycerofructose (specific schedule Mannitol 125ml every 12 hours, intravenously for 7 days; Glycerol Fructose 250ml once a day, intravenously for 3 days.) to reduce intracranial pressure for symptomatic treatment. Cranial magnetic resonance imaging was performed at Shanghai Integrated Traditional Chinese and Western Medicine Hospital Affiliated to Shanghai University of Traditional Chinese Medicine,and revealed multiple abnormal signals in the right cerebellar hemisphere and both cerebral hemispheres. Oral targeted therapy with anlotinib, Per Os,8mg from day1 to day14,every 3weeks was performed. 12 days following the patient’s intracranial hypertension.The patient received whole-brain and right-leg radiotherapy at Renji Hospital (Shanghai, China). Considering that carboplatin can enhance the sensitivity to radiotherapy and has fewer adverse reactions and greater nephrotoxicity than cisplatin, the cisplatin was replaced with carboplatin in the chemotherapy regimen, and a fourth course of immunotherapy + chemotherapy (375 mg cadonilimab, intravenously, on day 1 every 3 weeks; 500 mg carboplatin on day 1, intravenously, + 1,200 mg gemcitabine on days 1 and 8, intravenously, every 3 weeks) was administered. However, the patient developed severe bone marrow suppression, and blood tests revealed a hemoglobin concentration of 66 g/l(normal range120-175g/L). A 3-IU red blood cell suspension was administered to correct the anemia. The next day (D8), the patient received reduced-dose chemotherapy (1,000 mg gemcitabine).

In April 2023, the puncture biopsy sample admitted to the Cancer Hospital Affiliated to FuDan University (China, Shanghai) for pathological consultation and was diagnosed with differentially differentiated cancer (testicular tumor) consistent with SMARCB1/INI-1 deletion. The deletion of the SMARCB1/INI1 was confirmed by immunohistochemistry the following features can be observed: i) Tumor cell morphology shows diffuse distribution of atypic cells in tumor tissues, enlarged nuclei, increased nucleocytoplasmic ratio and an irregular nuclear membrane. The cells are pleomorphic, with distinct nucleoli and some cells show obvious nuclear division the normal testicular tissue structure is partially absent or deformed, and some normal testicular cells are scattered inside the tumor. In the tumor tissue, there is a fibrous tissue wrapping, and tumor cells invade and break through part of the envelope There is also the presence of cancer thrombi in the surrounding blood vessels.SMARCB1/INI-1 immunohistochemistry assays are used to assess the expression of INI-1 protein. In SMARCB1-deficient carcinoma of the testicle, the following features are commonly observed: In normal testicular tissue, INI-1 is usually uniformly expressed within the nucleus However, in tumors with deletion of SMARCB1, INI-1 expression is often markedly reduced or absent, manifesting as no or weak staining response in the nucleus. Immunohistochemical staining allows for clear visualization of the difference between INI-1-deleted tumor cells and normal cells. This absence can provide important clues to the differential diagnosis of tumors

**Figure 2:**
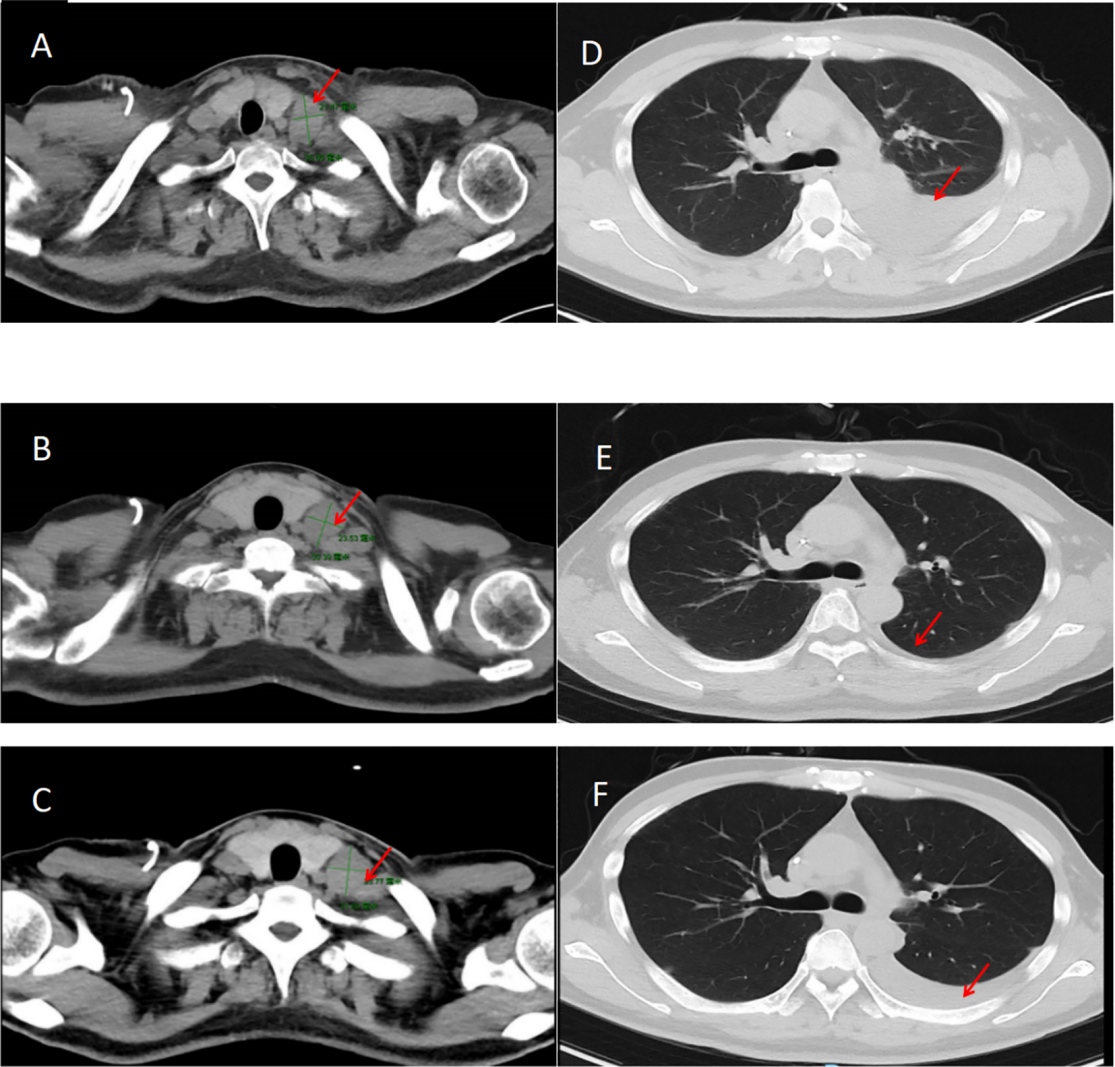
**(A)** Neck CT in January 2023 showing a supraclavicular lymph node measuring 21.47x35.09 mm. **(B)** CT of the neck in March 2023 showing the resolution of the supraclavicular lymph node involvement, and a size of ~23.53x30.39 mm. **(C)** Chest CT in May 2023 showing a supraclavicular lymph node size of ~23.77x31.69 mm. **(D)** CT of the chest in January 2023 showing a left-sided pleural effusion. **(E)** CT of the chest in March 2023 showing resolution of the pleural effusion. **(F)** CT of the chest in May 2023 showing that the pleural effusion is largely gone. CT, computed tomography.

The patient could not tolerate the severe bone marrow suppression caused by this chemotherapy regimen, so the regimen was changed to oxaliplatin and albumin-paclitaxel, which has fewer adverse reactions than the first- and second-generation platinum chemotherapy drugs. The first course of immunotherapy + chemotherapy (cadonilimab,375 mg, intravenously, on day 1 every 3 weeks; oxaliplatin,115 mg, intravenously,on day 1 every 3 weeks + albumin-paclitaxel 300 mg, intravenously, on day 1, every 3 weeks) was administered in May 2023. Later, due to a sharp decline in the patient’s physical condition, a second course of immunotherapy + reduced-dose chemotherapy (cadonilimab,375 mg, intravenously on day 1,every 3 weeks, oxaliplatin 100 mg, intravenously, on day 1 every 3 weeks + 200 mg oxaliplatin, intravenously, on day 1, every 3 weeks) was administered in June 2023. (**Figure 2** Comparison of CT results). Unfortunately, the patient ultimately passed away due to respiratory failure in July 2023.

## Discussion

SMARCB1-deficient carcinoma is a newly proposed gene-related malignant solid tumor with a clear genotype, which is a relatively well-diagnosed and independently classified tumor among the undifferentiated cancers of the head and neck. SMARCB1-deficient carcinoma involves a deletion of the SMARCB1 gene, also known as the INI-1 or SNF5 gene, which is a tumor suppressor gene located on chromosome 22q11.2 (37). The inactivation of this gene is associated with the pathogenesis of a variety of malignancies such as rhabdoid tumors and sinonasal carcinomas (38). In recent years, with the development of immunohistochemistry and molecular pathology, an increasing number of SMARCB1-deficient tumors have been reported and defined as SMARCB1-deficient tumors. Currently, SMARCB1/INI-1 - deficient tumors are classified into three types, including complete loss, mosaic expression, and reduced expression.hSNF5/INI1 gene in most cases, with decreased or absent expression at the RNA or protein level (39). The commonalities of the three types of SMARCB1/INI1 protein abnormalities are as follows: there is a variable proportion of “rhabdoid” cells, and different pathological types exhibit different modes of abnormal expression. The loss of INI1 (SMARCB1) protein expression is the result of biallelic inactivation involving the SMARCB1 gene locus, which can be caused by deletions and/or mutations. Missense mutations, nonsense mutations, splice site mutations, frameshift mutations, and in-frame deletions can all alter the synthesis status of the SMARCB1 protein (39). Studies have shown that SMARCB1 is a tumor suppressor gene *in vivo*, and SMARCB1 deficiency is observed in human malignant tumors such as T cell lymphoma rare rhabdoid tumors (40); SMARCB1 is involved in chromatin remodeling and transcriptional regulation, and is closely related to the processes of cell differentiation and cell proliferation (40). Therefore, SMARCB1 deficiency is closely related to the occurrence of tumors (41). Conditional knockout of SMARCB1 leads to cancer formation in adult mice. SMARCB1/INI1 gene loss was first detected in soft-tissue tumors (42), such as renal and extrarenal malignant rhabdoid tumors, and atypical teratoid/rhabdoid tumors. Therefore, SMARCB1/INI1 gene mution has become a characteristic diagnostic indicator. Modern diagnostic technology has identified a type of cancer called SMARCB1-deficient cancer. Unlike other types of cancer, this cancer usually has a stable genome and only harbors a single gene mutation of SMARCB1 (43). Complete loss of SMARCB1/INI1 expression is common in children and young adults, but such tumors are rarely observed in adults. The diagnosis of SMARCB1/INI1-deficient tumors is difficult due to their polymorphic variability (44). SMARCB1-deficient tumors generally have a poor prognosis, are often widely metastatic at diagnosis (6), are resistant to cytotoxic chemotherapy and have a high mortality rate (45). Regardless of histological classification, the median patient survival time is only 6 months (46). Therefore, the early differential diagnosis of this type of cancer is particularly important.

**Figure 3:**
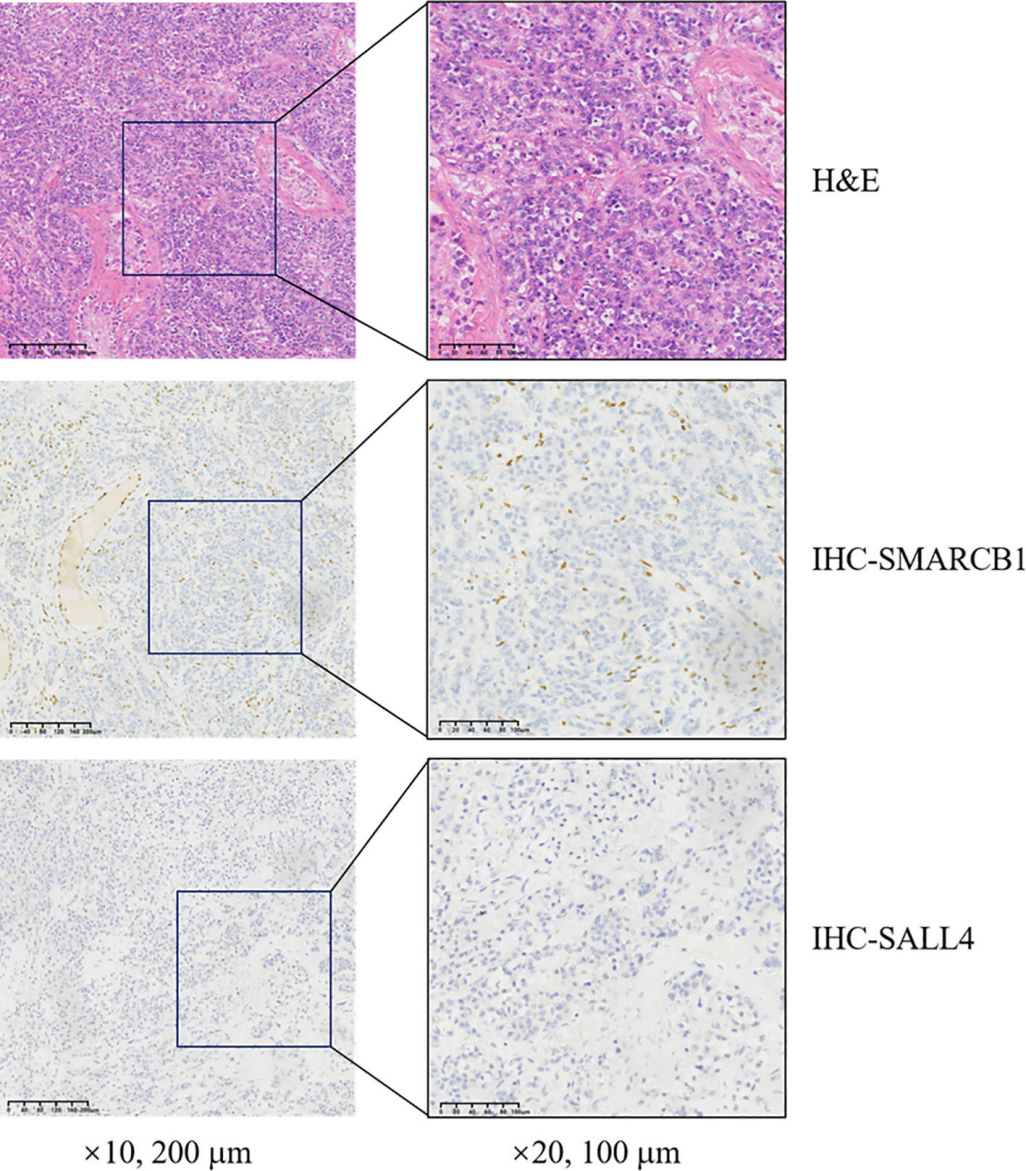
Histopathological analysis of resected tissue demonstrating SMARCB1 deletion. H&E, hematoxylin and eosin; SMARCB1, switch/sucrose non-fermentable-related matrix-associated actin-dependent regulator of chromatin subfamily B member 1; SALL4, sal-like protein 4; IHC, immunohistochemistry.

**Figure 4:**
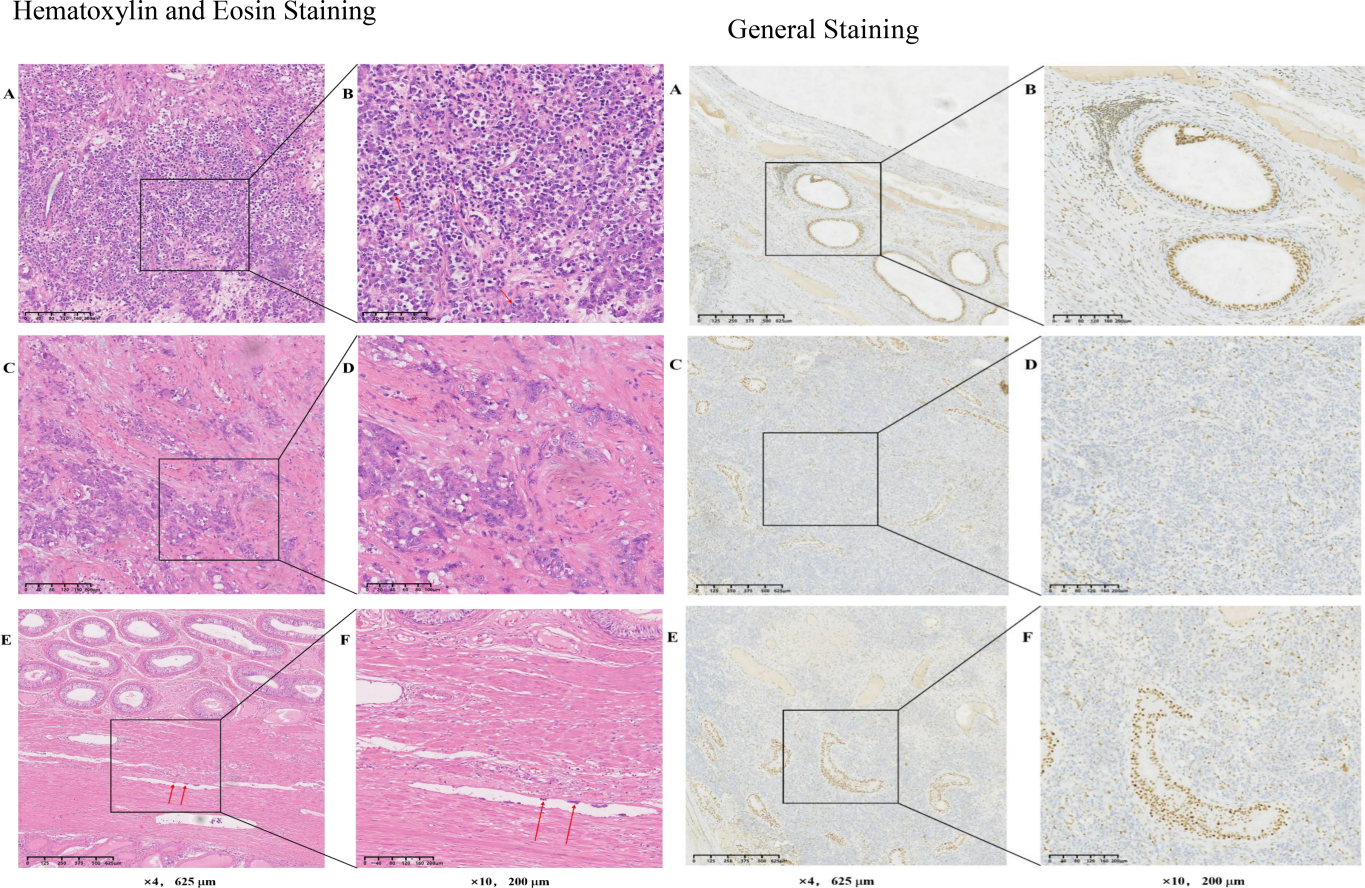
Hematoxylin and Eosin Staining:Hematoxylin and Eosin Staining **(A)** The tumor cells are pleomorphic, with distinct nucleoli and some tumor cells showing obvious nuclear division. **(B)** Image **(A)** at increased magnification. **(C)** Fibrous tissue wrapping is present, and tumor cells invade and break through part of the envelope. **(D)** Image **(C)** at increased magnification. **(E, F)** Thrombus in the blood vessels. **(F)** Image **(E)** at increased magnification.**G**eneral Staining**(A)** Normal testicular tissue expressing INI-1. **(B)** Image **(A)** at increased magnification. **(C)** Weak staining response in the nucleus, in the tumors with deletion of SMRCB1. **(D)** Image **(C)** at increased magnification. **(E)** INI-1-deleted tumor cells and normal cells in immunohistochemical staining. **(F)** Image **(E)** at increased magnification. SMRCB1/INI, switch/sucrose non-fermentable-related matrix-associated actin-dependent regulator of chromatin subfamily B member 1.

There are aspects worth reflecting on in the treatment of this case. Although the postoperative immunohistochemistry showed (**Figures 3**, **4**) the loss of SMARCB1/INI-1 expression and the negative expression of OCT4, considering the location of the tumor and the patient’s age, the patient still received adjuvant chemotherapy for testicular seminoma after surgery. However, the treatment outcome was not satisfactory, and progression occurred shortly after the operation.When lymph node metastasis occurred, another puncture biopsy sample was sent to the Affiliated Cancer Hospital of Fudan University for pathological consultation, and the sample was confirmed to be a poorly differentiated carcinoma (testicular tumor) consistent with SMARCB1/INI-1 deletion.

In the differential diagnosis between SMARCB1/INI-1-deficient tumors and testicular seminoma in this case,The negative expression of OCT4 is a breakthrough point in the diagnosis of testicular seminoma in this case. A testicular seminoma is a malignant tumor originating from the testicular germ cells that accounts for 90-95% of testicular malignant tumors. The main clinical symptoms are testicular enlargement and heaviness or pain (47). In 1-3% of patients with testicular seminoma, the first symptom is tumor metastasis, with retroperitoneal metastasis being the most common (48). Seminomas are often localized lesions with slow progression and high sensitivity to radiotherapy. For early stage seminomas, surgical removal of the diseased testicles and adjuvant radiotherapy and chemotherapy can often cure the disease.Seminomas are common malignant tumors in clinical practice; they are malignant germ cell tumors that originate from primordial germ cells and gonocytes during early embryonic development. However, Seminomas are less malignant and more sensitive to radiotherapy compared with the SMARCB1-deficient tumor,and patients generally have a good prognosis (49). However, OCT4 is expressed positively in all cases of testicular seminoma.

OCT4 is a key transcription factor that belongs to the POU domain transcription factor family. It plays an important role in maintaining the pluripotency of embryonic stem cells (ESCs) and the characteristics of cancer stem cells (50). In reproductive system tumors, OCT4 is mainly expressed in the germ cell components of seminomas, embryonal carcinomas, dysgerminomas, and gonadoblastomas (51). In particular, the positivity rate of OCT4 in seminomas is as high as 100%, while it is negative in normal testicular tissue (52). Therefore, OCT4 staining is routinely used for the diagnosis of primary germ cell tumors in the testis and other locations (53). The expression of OCT4 is closely related to tumor invasion and metastasis, and may lead to poor prognosis in patients (54). Oct4 transcriptionally activates the expression of NEAT1 and MALAT1 to accelerate the progression of lung cancer (55). Oct4 promotes the malignant phenotype of cervical cancer cells and the polarization of M2 macrophages and cervical cancer metastasis by activating the p38 pathway (56). The postoperative pathology of the patient reported in this case showed that OCT4 expression was negative. On one hand, this result can rule out the diagnosis of seminoma, as OCT4 is typically positive in seminomas. On the other hand, the negative OCT4 result indirectly indicates that OCT4 is not the cause of the rapid progression of the patient’s condition. This suggests that the patient’s tumor may have other unique molecular characteristics or biological mechanisms that lead to its high invasiveness and rapid progression. Therefore, the focus of this article is on SMARCB1-deficient tumors.

The patient with a SMARCB1-deficient testicular tumor reported in the present study experienced disease progression and multiple metastases only 4 months after the surgery, which shows that tumors with SMARCB1 deficiency are highly malignant and invasive. PD-L1 expression levels and drug sensitivity information was obtained for the patient through lymph node puncture to provide a reliable basis for further treatment. Based on the patient’s drug sensitivity information, a chemotherapy regimen of gemcitabine combined with cisplatin was selected. Based on the patient’s performance status, anlotinib, a small molecule multitarget tyrosine kinase inhibitor that can inhibit kinases such as VEGFR, PDGFR, FGFR and c-Kit, was also selected. The drug can not only inhibit tumor angiogenesis, but also inhibit tumor growth.

**Figure 5:**
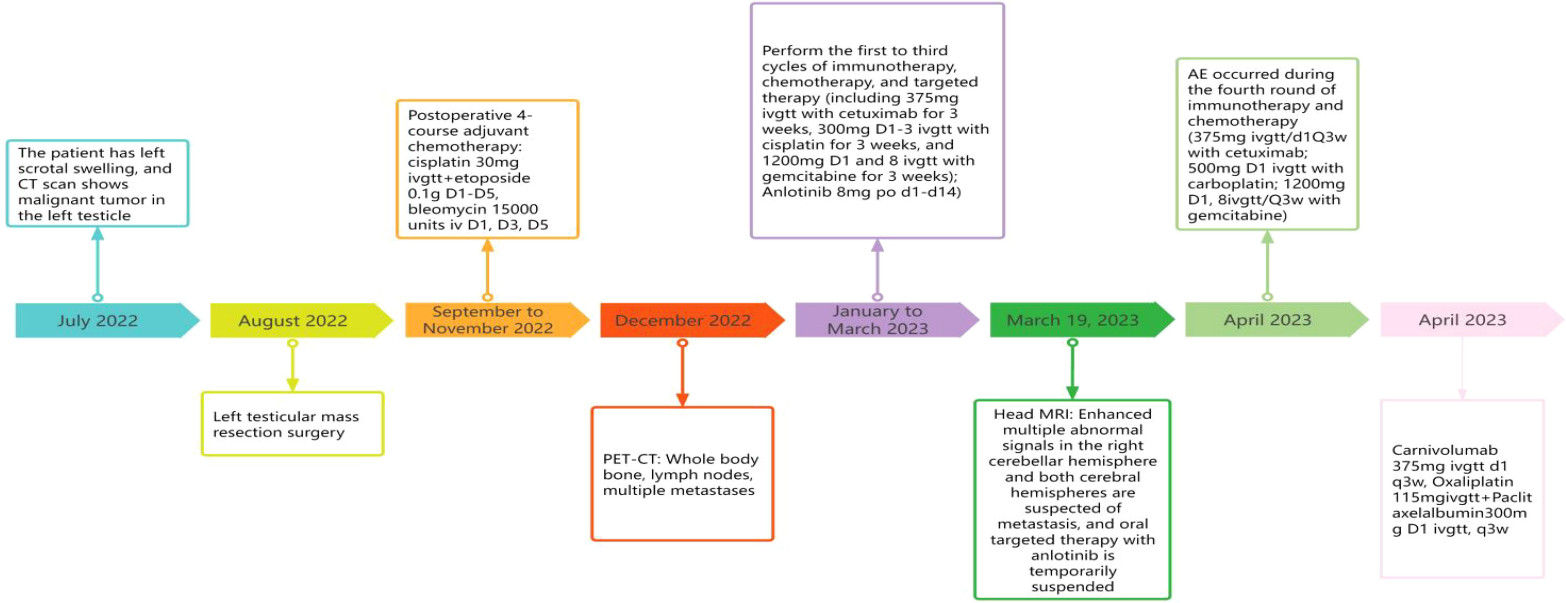
The overall treatment of the case.

**Figure 6:**
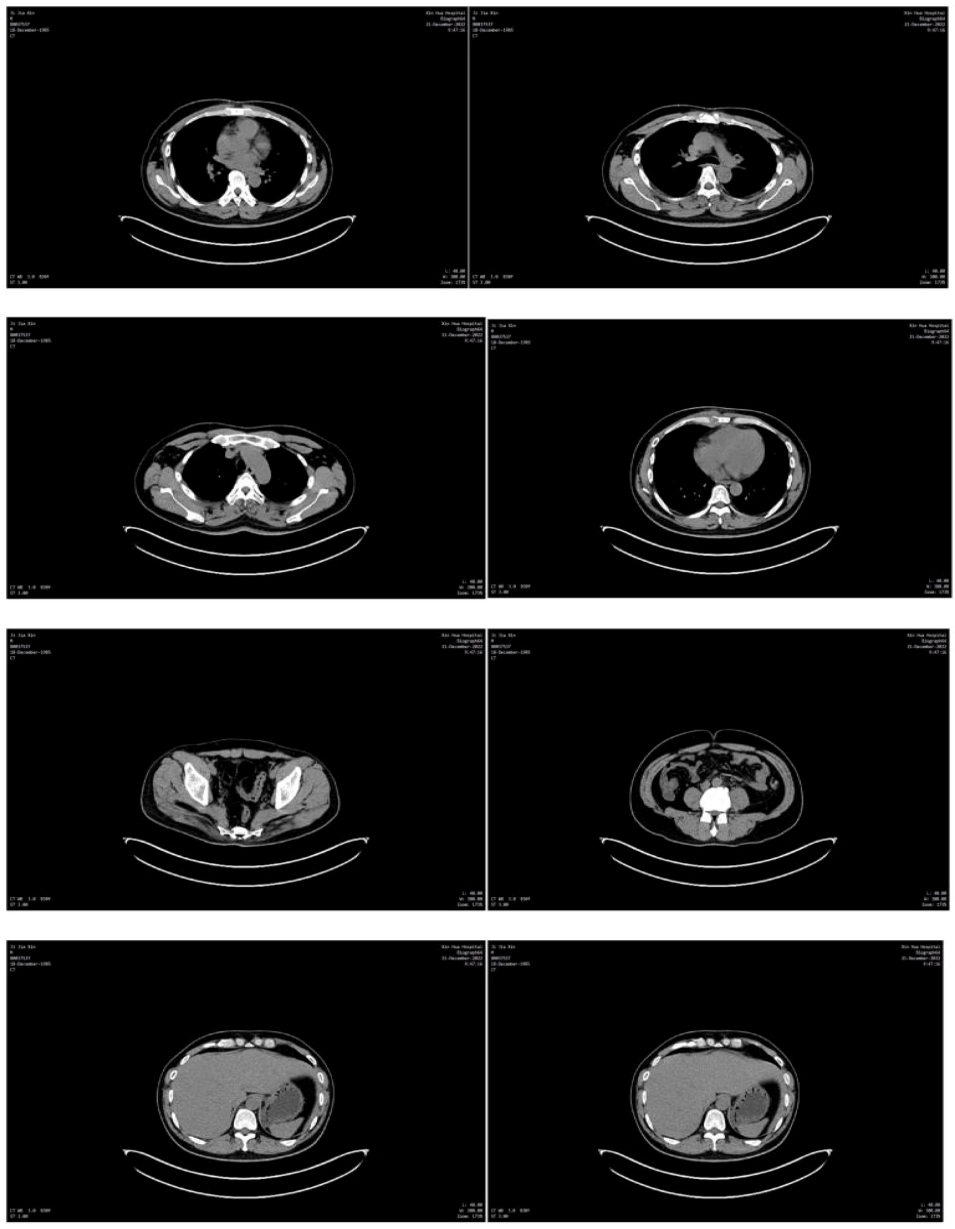
Multiple bone metastases throughout the body.

Considering that SMARCB1-deficient tumors exhibit clonal expansion of T-cell subsets and have higher tumor-specific immune responses, in this case report, the patient had a Tumor Proportion Score (TPS) of 30%, which is considered a moderate level of expression, and a Combined Positive Score (CPS) of 40%. These indicators suggested a potential benefit from immunotherapy. Therefore, we explored the use of cadonilimab for immunotherapy in this patient. Cadonilimab is a bispecific antibody that targets both PD-1 (Programmed Death-1) and CTLA-4 (Cytotoxic T-Lymphocyte-Associated Protein 4) receptors. which can act on both programmed cell death protein 1 (PD-1) and cytotoxic T lymphocyte-associated antigen 4 (CTLA-4) receptors, and can multivalently bind to T cells that coexpress PD-1 and CTLA-4 in the tumor microenvironment. While blocking the PD-1 signaling pathway, it can also inhibit the CTLA-4 signaling pathway, thereby enhancing the immune activity of T cells and ultimately improving their antitumor ability (57). The results of a phase II clinical study of cadonilimab (AK104-201) showed that regardless of PD-L1 expression status, the objective response rate (ORR) of the entire population reached 33%; subgroup analysis revealed that the ORR of patients with positive PD-L1 expression (CPS ≥1) was as high as 43.8% (2.5 times that of Pembrolizumab), and the ORR of patients with negative PD-L1 expression was 16.7%, which was close to the ORR of patients with positive PD-L1 expression(which refers to CPS ≥1) to Pembrolizumab (17). However, despite the use of cadonilimab, the patient’s condition still progressed rapidly. This may be related to the patient’s relatively low Tumor Proportion Score (TPS) and low Tumor Mutational Burden (TMB).Given the highly aggressive clinical course of SWI/SNF-deficient malignancies, systemic chemotherapy has not yet demonstrated success to date, and thus no satisfactory systemic chemotherapy has been established for these patients (58). Based on previous studies, cisplatin has demonstrated good therapeutic effects in the majority (80%) of patients with metastatic germ cell tumors (GCTs), and can even achieve continuous complete remission. Gemcitabine also has good anticancer activity in advanced GCTs. Therefore, we chose the GP regimen (gemcitabine plus cisplatin) as the second-line treatment for the patient after recurrence (59). However, testicular cancer can be resistant to cisplatin, and approximately 10-20% of patients with metastatic testicular cancer (TC) cannot be cured by cisplatin-based chemotherapy. Mechanisms of resistance include the downregulation of OCT4 and the failure to induce PUMA and NOXA, as well as elevated levels of MDM2 and hyperactivity of the PI3K/AKT/mTOR pathway. In this case, the patient also developed cisplatin resistance, which led to disease progression (60). Unfortunately, after substituting cisplatin with carboplatin, the patient experienced severe bone marrow suppression, which forced another change in the chemotherapy regimen (**Figures 5**, **6**). Additionally, the clinical course of SWI/SNF-deficient malignancies is highly aggressive, leading to widespread dissemination of the disease at or shortly after diagnosis and resulting in death shortly after diagnosis, despite curative treatment intent.

Since the SMARCB1 gene has only been gradually discovered in the past decade or so, the literature on tumors with SMARCB1 deficiency is very limited. Currently, clinical research in this area is primarily conducted through case reports. There are only basket studies and a few Phase II clinical trials available. The exploration of treatment strategies is mainly focused on preclinical research. Therefore, in the subsequent sections of this article, we will review the molecular functions of SMARCB1, the tumor occurrence mechanisms mediated by it, and related signaling pathways to identify potential therapeutic approaches (**Table 1**).

**Table 1:** Key diagnostic and treatment.

Pathway	Treatment Category	Specific Therapy	Mechanism of Action	References
ErbB2-EGFR pathway	Targeted Therapy	Lapatinib	Inhibits the ErbB2 and EGFR signaling pathways, suppressing tumor cell proliferation	(14)
FGFR pathway	Targeted Therapy	NVP-BGJ398	Inhibits the FGFR signaling pathway, suppressing tumor cell proliferation	(15)
PDGFRα-FGFR1 pathway	Targeted Therapy	Ponatinib	Inhibits the PDGFRα and FGFR1 signaling pathways, suppressing tumor cell proliferation	(16)
PD-1, CTLA-4 signaling pathways	Immunotherapy	Pembrolizumab, Cadonilimab	Blocks PD-1 and CTLA-4 signaling pathways to enhance T-cell	(17–20)
PD-1 signaling pathway	Immunotherapy	Tislelizumab	Blocks the PD-1 signaling pathway to enhance T-cell antitumor activity	(21)
PRC2-EZH2 pathway	Epigenetic Therapy	Tazemetostat	Inhibits EZH2 activity, blocks PRC2-mediated gene silencing, and restores tumor suppressor gene expression	(22–26)
GSK-3β, AMPK/mTORC1/ULK1 pathways	Epigenetic Therapy	CHIR99021	Inhibits GSK-3β activity, activates the AMPK/mTORC1/ULK1 pathway, inducing cell cycle arrest and autophagy	(27)
IL6/JAK/STAT3 pathway	Pathway Targeting	IL6/JAK/STAT3 inhibitors (e.g., TTI-101)	Inhibits the IL6/JAK/STAT3 signaling pathway, reducing tumor cell proliferation	(28)
Hedgehog-GLI1 pathway	Pathway Targeting	Hedgehog pathway inhibitors	Inhibits the Hedgehog signaling pathway, reducing GLI1 activity and suppressing tumor cell proliferation	(29)
WNT/β-catenin pathway	Pathway Targeting	WNT/β-catenin pathway inhibitors	Inhibits the WNT signaling pathway, reducing β-catenin activity and suppressing tumor cell proliferation	(30)
/	Other	DCAF5 targeting	Depletes DCAF5 to stabilize the SWI/SNF complex, restoring normal gene expression	(31)
/	Other	scL-SMARCB1 nanocomplex	Delivers wild-type SMARCB1 gene, restores SMARCB1 expression, and inhibits tumor proliferation	(32)
/	Other	PHF6 targeting	Targets PHF6 to restore the stability and function of the SWI/SNF complex	(33)
/	Other	BRD9 inhibition	Degrades BRD9 to disrupt the SWI/SNF complex, inhibiting tumor growth	(34, 35)
MYC signaling pathway	Other	Proteasome inhibition or autophagy inhibition	Targets MYC-activated cellular programs due to SMARCB1 deficiency, inhibiting tumor growth	(36)

### Polycomb pathway and EZH2 inhibitors

In addition, enhancer of zeste homolog 2 (EZH2) inhibitors are new drugs for the treatment of cancers lacking SMARCB1, and preclinical studies have shown that they have the potential to modulate tumor immunogenicity and antitumor immune responses (57). Polycomb repressive complex 2 (PRC2), a component of which is regulated by the SWI/SNF complex, also contains SMARCB1. Thus, loss of SMARCB1 leads to dysregulation of PRC2- and EZH2-mediated gene silencing, thereby promoting retention of the stem cell phenotype and tumorigenesis (61, 62).

Polycomb group (PcG) proteins are a group of transcriptional repressors that regulate the expression of target genes through chromatin modifications. They include PRC1 (Polycomb repressive complex 1) and PRC2 (Polycomb repressive complex 2).

(63). PcG proteins play a crucial role in embryonic development by regulating the expression of cell cycle-related genes, thereby influencing cell proliferation and differentiation. EZH2 is the catalytic subunit of the PRC2 complex, which is responsible for adding the epigenetic silencing mark H3K27me3. Activation of EZH2 upregulates oncogenic pathways, including the c-Myc signaling pathway, the Sonic hedgehog (Shh) signaling pathway, and the Wnt/β-catenin signaling pathway, while also suppressing the transcription of tumor suppressor genes (64). EZH2 regulates the cell cycle process. Dysregulation of EZH2 can accelerate cell proliferation and extend cell survival, which may lead to oncogenesis and cancer progression (65). The overexpression and activity of EZH2 are particularly prominent in tumors with SMARCB1 inactivation, making it an effective therapeutic target (66). Recent studies have confirmed that tazemetostat, a selective EZH2 inhibitor, induces effective anti-proliferative and anti-tumor effects in cell lines and xenografts of small-cell carcinoma of the ovary, hypercalcemic type (SCCOHT), which are deficient in both SMARCA2 and SMARCA4 (22). The mechanism may involve the inhibition of neural differentiation genes, cell cycle inhibitors, and tumor suppressor factors by EZH2, while also reducing the expression of GLI1, Patch1, MYC.Tazemetostat is the first-in-class oral EZH2 inhibitor approved by the U.S. Food and Drug Administration (FDA) for the treatment of SMARCB1-deficient epithelioid sarcoma (23). Tazemetostat has shown good objective response rates in several small-sample clinical studies (24, 25). In an open-label, phase 2 basket study, 62 patients with epithelioid sarcoma characterized by INI1/SMARCB1 loss were enrolled. The results suggested that tazemetostat may improve the prognosis of patients with advanced epithelioid sarcoma, with a median progression-free survival of 5.5 months (95% CI 3.4-5.9) and a median overall survival of 19.0 months (11.0-not estimable) (26). Dual inhibition of EZH1/2 is also one of the promising therapeutic approaches for SMARCB1-deficient tumors worth exploring in future clinical practice.EZH1 and EZH2 contribute to the growth of MRT (Malignant Rhabdoid Tumor) cells and H3K27 methylation. Depletion or selective inhibition of EZH2 leads to compensatory upregulation of EZH1 expression, whereas depletion of EZH1 enhances the effect of EZH2 inhibition.Preclinical studies have found that dual inhibitors of EZH1/2 significantly suppress the growth of MRT (Malignant Rhabdoid Tumor) cells, specifically manifested by a reduction in the accumulation of H3K27me3 at the CDKN2A locus, which is one of the targets of EZH1/2. Dual inhibition of EZH1/2 *in vivo* completely inhibits tumor growth without significant adverse effects (67).

### IL6/JAK/STAT3 pathway and STAT3 inhibitor

One study showed that low SMARCB1 expression promoted Bladder cancer growth by activating STAT3; therefore, targeting the IL6/JAK/STAT3 pathway is a potential treatment for SMARCB1-deficient tumors (28). The STAT3 inhibitor TTI-101 was shown to effectively inhibit tumor growth in SMARCB1-deficient BLCA patient-derived xenograft mice without significant weight loss, providing a strong preclinical rationale for using TTI-101 as a therapeutic strategy for SMARCB1-deficient BLCA.

### Hedgehog pathway and GLI1

Aberrant activation of the Hedgehog (Hh) pathway can drive tumorigenesis. Mutations in the PTCH1 gene (encoding Patched-1 receptor) and the SMO gene (encoding the Smoothened homolog) lead to abnormal pathway activation, resulting in medulloblastoma and basal cell carcinoma. Studies have shown that Snf5 is localized to the promoter regulated by Gli1, and loss of Snf5 leads to activation of the Hh-Gli pathway. Conversely, re-expression of SNF5 in MRT (malignant rhabdoid tumor) cells inhibits GLI1. Consistent with this, primary malignant rhabdoid tumors exhibit a gene expression profile of Hh-Gli activation, indicating that GLI1 drives the growth of SNF5-deficient malignant rhabdoid tumor cells both *in vitro* and *in vivo*. Therefore, targeting the abnormal activation of GLI1 is a potential therapeutic strategy for SMARCB1 tumors (29).

### Tyrosine kinase inhibitors

Tyrosine kinase inhibitors are a class of drugs that disrupt cancer cell signaling and proliferation by inhibiting the activity of tyrosine kinases. They are characterized by high selectivity, low toxicity, and minimal side effects. Lapatinib is a tyrosine kinase inhibitor that targets both ErbB2 (HER2) and EGFR (HER1). By inhibiting the ErbB2-EGFR pathway, lapatinib can effectively suppress the activity of central nervous system atypical teratoid/rhabdoid tumor (CNS ATRT) tumor cells harboring INI1 mutations, both *in vitro* and *in vivo (* 14). Additional studies have shown that in MRT cells, the absence of SMARCB1 is associated with upregulation of FGFR1, and the levels of FGFR1 are regulated by SMARCB1 (15, 68). Loss of SNF5 function is associated with increased expression of fibroblast growth factor receptor (FGFR) in MRT cell lines and primary tumors. Moreover, re-expression of SNF5 in MRT cells leads to significant suppression of FGFR expression. Conversely, siRNA-mediated impairment of SWI/SNF function results in elevated levels of FGFR2 in human fibroblasts. *In vivo* studies have shown that treatment with the selective FGFR inhibitor NVP-BGJ398 can block the progression of MRT in mouse models.The efficacy of the dual-target inhibitor ponatinib in SMARCB1-deficient tumors remains controversial (15). Some studies have shown that ponatinib can inhibit the activity of PDGFRα and FGFR1, thereby suppressing the phosphorylation of AKT and ERK1/2, leading to a synergistic cytotoxic effect in MRT cells. *In vitro* experiments have demonstrated that ponatinib, as a single agent, exhibits certain therapeutic effects on MRT (16). In AT/RT cell lines, inhibitors targeting PDGFRα and FGFR have shown limited efficacy in reducing cell viability. This may be attributed to the complexity of SMARCB1-deficient tumors (14). Glycogen synthase kinase-3β (GSK-3β) is a serine/threonine kinase that phosphorylates a broad range of substrates and plays a significant role in the development and progression of tumors (69). In embryonic stem (ES) cells, selective inhibition of GSK-3α/β by CHIR99021 induces cell cycle arrest, mitotic catastrophe (MC), and autophagy activation mediated by the AMPK/mTORC1/ULK1 pathway, resulting in decreased cell proliferation (27). Despite the substantial progress in the clinical application of tyrosine kinase inhibitors for a variety of tumors, the development of targeted therapies specifically addressing SMARCB1-deficient malignancies remains an area of significant unmet need and active investigation.

### WNT/β-catenin pathway

Defects in SMARCB1/INI1 lead to aberrant activation of the WNT signaling pathway, resulting in phenotypic defects consistent with overexpression of WNT/β-catenin (30).Studies have shown that the loss of Snf5 gene copy leads to excessive activation of β-catenin, and SNF5 is crucial for regulating the expression of Wnt/β-catenin pathway targets. However, to date, no studies have investigated β-catenin inhibitors targeting SMARCB1-deficient tumors. Gene set enrichment analysis has revealed that, compared with normal cerebellum, Wnt targets are elevated in SNF5-deficient rhabdoid tumors, and the abnormal activation of β-catenin target genes occurs independently of canonical Wnt pathway activation (30).

### Immune checkpoint inhibitors

The question of whether SMARCB1-deficient tumors can benefit from immunotherapy remains controversial. On one hand, SMARCB1-deficient tumors often exhibit low tumor mutation burden, which may render them less responsive to immunotherapy.In a phase II clinical trial that reported the first prospective study of renal medullary carcinoma (RMC) with loss of SMARCB1 protein (also known as INI-1, hSNF5, or BAF47), results showed no evidence of clinical activity of pembrolizumab in RMC patients, regardless of PD-L1 or tumor-infiltrating lymphocyte (TIL) levels (18). On the other hand, immunotherapy has demonstrated promising responses in some reported clinical cases (19, 20). Forrest et al. (43) used PD-L1 to treat three children with SMARCB1 mutations. One patient was an 18-year-old man who was diagnosed with epithelioid sarcoma. In total, 40% of the tumor cells were PD-L1^+^. The patient received 18 cycles of pembrolizumab treatment, after which palpable lymph node tumors appeared. Talimogene laherparepvec and systemic pembrolizumab treatment were administered. After two cycles of the treatment, the size of the lesion was significantly reduced by 18.5%, and after four cycles, the lesion had basically disappeared (70). Some scholars have proposed that first-line ABCP (atezolizumab, bevacizumab, paclitaxel, and carboplatin) treatment demonstrates durable efficacy in SMARCA4-deficient thoracic sarcoma (SMARCA4-DTS), regardless of the degree of PD-L1 expression (71). In preclinical studies,Leruste and colleagues demonstrated that, despite the very simple genome and extremely low mutation burden of malignant rhabdoid tumors (MRTs), they are immunogenic and may have good outcomes from immunotherapy (72). Msaouel et al. found that the dsDNA-sensing cGAS/STING pathway is activated and associated with enhanced tumor immunogenicity. They suggested that the loss of SMARCB1 leads to increased replicative stress due to the activation of the c-MYC pathway, followed by the activation of cell cycle checkpoints and increased DNA damage (73). There are also clinical cases showing that patients with SMARCB1-deficient tumors have benefited from immunotherapy. In one study, a patient with advanced refractory SMARCB1-deficient epithelioid sarcoma achieved complete remission after receiving combination treatment with ipilimumab and nivolumab (74); another patient with rectal SMARCB1-deficient undifferentiated carcinoma had complete disappearance of tumor lesions 8 months after receiving the PD-1 inhibitor tislelizumab following recurrence post-surgery (21)Given the limitations of prior studies on immunotherapy for SMARCB1-deficient tumors, which have often been small-scale or case-based, future research necessitates the conduct of larger-scale, multicenter clinical trials to more accurately assess the efficacy and safety of immunotherapy in this context.

### Other promising way

A recent study published in *Nature* utilized a genome-wide CRISPR screening to identify vulnerabilities in SMARCB1-mutant tumor cell lines. The study found that rhabdoid tumors (RT), which are highly aggressive due to the loss of the key tumor suppressor protein SMARCB1, could be suppressed by the depletion of the quality control protein DCAF5. This suggests that targeting DCAF5 may reverse the cancerous state.DCAF5 has a quality control function for the SWI/SNF complex and promotes the degradation of incompletely assembled SWI/SNF complexes in the absence of SMARCB1. Upon DCAF5 depletion, SMARCB1-deficient SWI/SNF complexes re-accumulate, bind to target loci, and restore SWI/SNF-mediated gene expression to levels sufficient to reverse the cancerous state, including *in vivo*. Therefore, the cancer is not caused by the loss of SMARCB1 function per se, but rather by DCAF5-mediated degradation of the SWI/SNF complex. Targeting DCAF5 to stabilize the SWI/SNF complex thus emerges as a potential strategy to inhibit SMARCB1-mutant cancers<sup>70.</sup>Recent studies have explored the therapeutic potential of nanoparticle-based drug delivery systems to expand treatment options for SMARCB1-deficient tumors. The scL-SMARCB1 nanocomplex is an immunolipid nanoparticle modified with a transferrin receptor single-chain antibody fragment (TfRscFv), which serves as the targeting moiety. TfRscFv enables the efficient delivery of a plasmid containing the human wild-type SMARCB1 gene to tumor cells.The scL-SMARCB1 nanocomplex restores SMARCB1 expression, which in turn inhibits the proliferation of ATRT (atypical teratoid/rhabdoid tumor) cells and induces senescence and apoptosis. Systemic administration of the scL-SMARCB1 nanocomplex as a monotherapy demonstrated antitumor efficacy in mice bearing ATRT xenografts, with the exogenous expression of SMARCB1 modulating MYC target genes. When combined with cisplatin-based chemotherapy or radiation, the scL-SMARCB1 nanocomplex exhibited enhanced antitumor efficacy, significantly improving the survival of mice with ATRT (32). In SMARCB1-deficient tumors, simultaneous targeting of CBP/p300 inhibition can indeed downregulate the transcription of KREMEN2, which further leads to the monomerization of KREMEN1, inducing apoptosis and thereby suppressing anti-apoptotic signaling pathways. Compared with other inhibitors, this approach shows higher selectivity and efficacy than tazemetostat (75). There have also been reports that the occurrence of SMARCB1-deficient tumors is associated with PHF6. PHF6 is an X-chromosome-encoded protein that contains two evolutionarily conserved atypical PHD finger domains, which are involved in protein-protein interactions related to epigenetic regulation. PHF6 co-localizes with the SWI/SNF complex at promoters, which is crucial for maintaining active chromatin states (76). In the absence of SMARCB1, the loss of PHF6 disrupts the recruitment and stability of residual SWI/SNF complex members, leading to the loss of active chromatin at promoters and stalling of RNA polymerase II. Therefore, targeting PHF6 may offer new therapeutic opportunities for SMARCB1-mutant cancers (33). Bromodomain-containing protein 9 (BRD9) is a subunit of the SWI/SNF (BAF) chromatin remodeling complex. Small-molecule BRD9 inhibitors have been shown to reduce the proliferation of SMARCB1-deficient rhabdoid tumor cells *in vitro* and induce G1 cell cycle arrest. Moreover, these inhibitors exhibit synergistic effects when combined with cisplatin and doxorubicin, significantly inhibiting the growth of rhabdoid tumor (RT) cells (34). Recent studies have identified a potent PROTAC degrader of BRD9, CW-3308, which achieves 91% oral bioavailability in mice. A single oral dose of CW-3308 efficiently reduces BRD9 protein levels by >90% in synovial sarcoma HS-SY-II xenograft tumor tissue. This degradation of BRD9 significantly inhibits the growth of HS-SY-II xenograft tumors in mice (35). Loss of SMARCB1 activates the transcription factor MYC, leading to significant upregulation of protein anabolism (77). SMARCB1-deficient mice develop endoplasmic reticulum stress and cytoplasmic protein aggregates, which may be due to activation of MYC-p19-regulated cellular programs through SMARCB1. Therefore, *in vitro* and *in vivo* malignant rhabdoid tumor and renal medullary carcinoma models respond well to treatment with proteasome inhibitors or autophagy inhibition (36).

In summary, to date, no SMARCB1-deficient testicular malignancies have been reported, and there is a lack of systematic diagnostic and treatment options for this disease the treatment of SMARCB1-deficient tumors is confronted with multiple challenges, including difficult diagnosis, high aggressiveness, high resistance to therapy, and a lack of effective treatment options. Future research needs to further explore the molecular mechanisms underlying these tumors, develop more effective diagnostic biomarkers and therapeutic targets, in order to improve the prognosis and quality of life for patients with these rare malignancies. Limitations of the present study include the fact that the CT imaging report and images could not be obtained from the initial onset before surgery, due to the simple structure of the testicles, the patient only underwent an ultrasound examination before surgery.

## Data Availability

The original contributions presented in the study are included in the article/**Supplementary Material**. Further inquiries can be directed to the corresponding authors.
