# Supplementary figures and images for: SMARCB1-deficient poorly differentiated testicular carcinoma: a case report
Source: Front Oncol. 2025 Mar 6;15:1554352. doi: 10.3389/fonc.2025.1554352 (PMC11922841; doi:10.3389/fonc.2025.1554352)

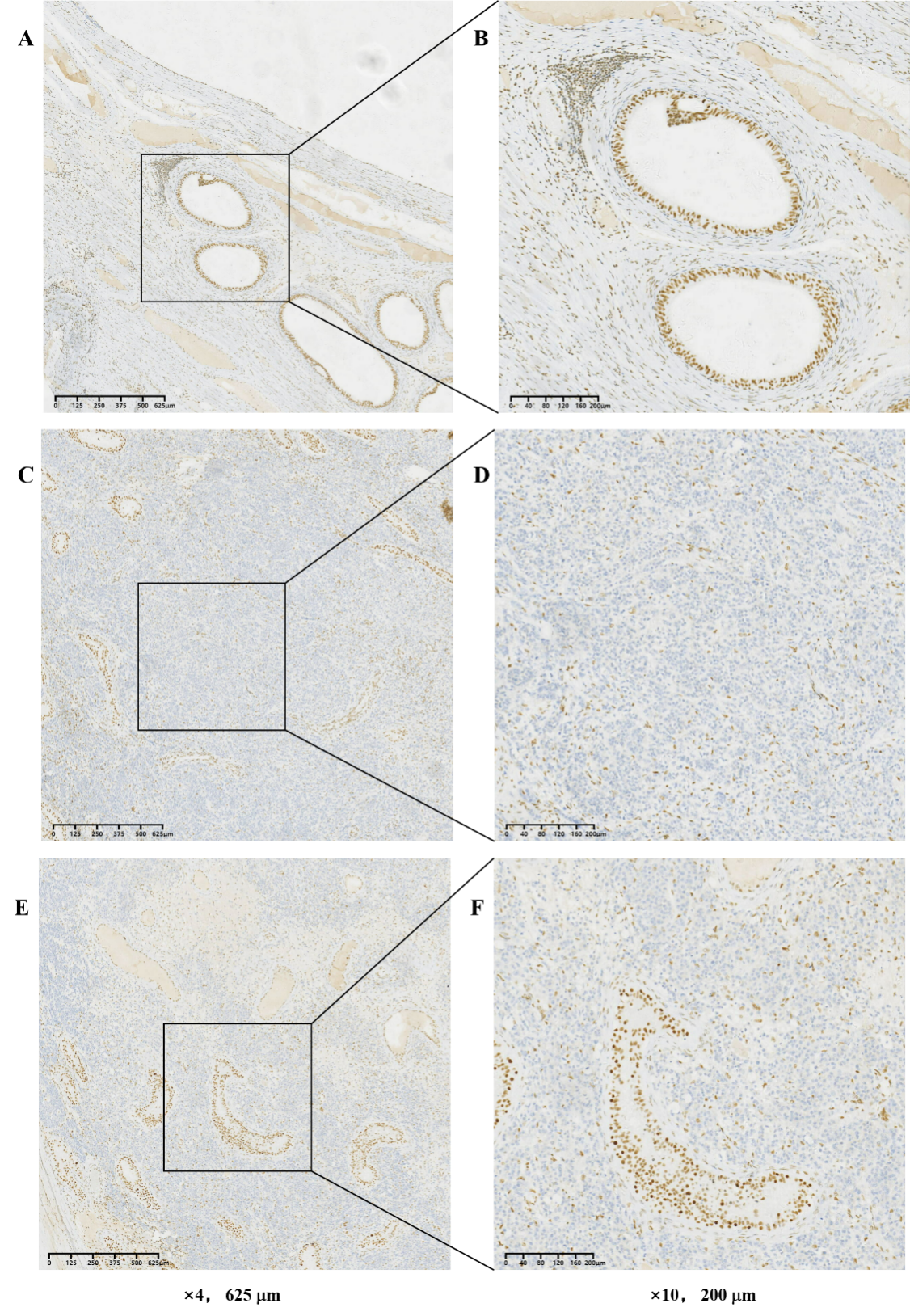

Supplement: Supplementary file 1 [file Image1.tif]
