# Supplementary material for: SMARCB1-deficient poorly differentiated testicular carcinoma: a case report
Source: Front Oncol. 2025 Mar 6;15:1554352. doi: 10.3389/fonc.2025.1554352 (PMC11922841; doi:10.3389/fonc.2025.1554352)
Supplement: Supplementary file 2 [file Table1.doc]

Preoperative B-ultrasound


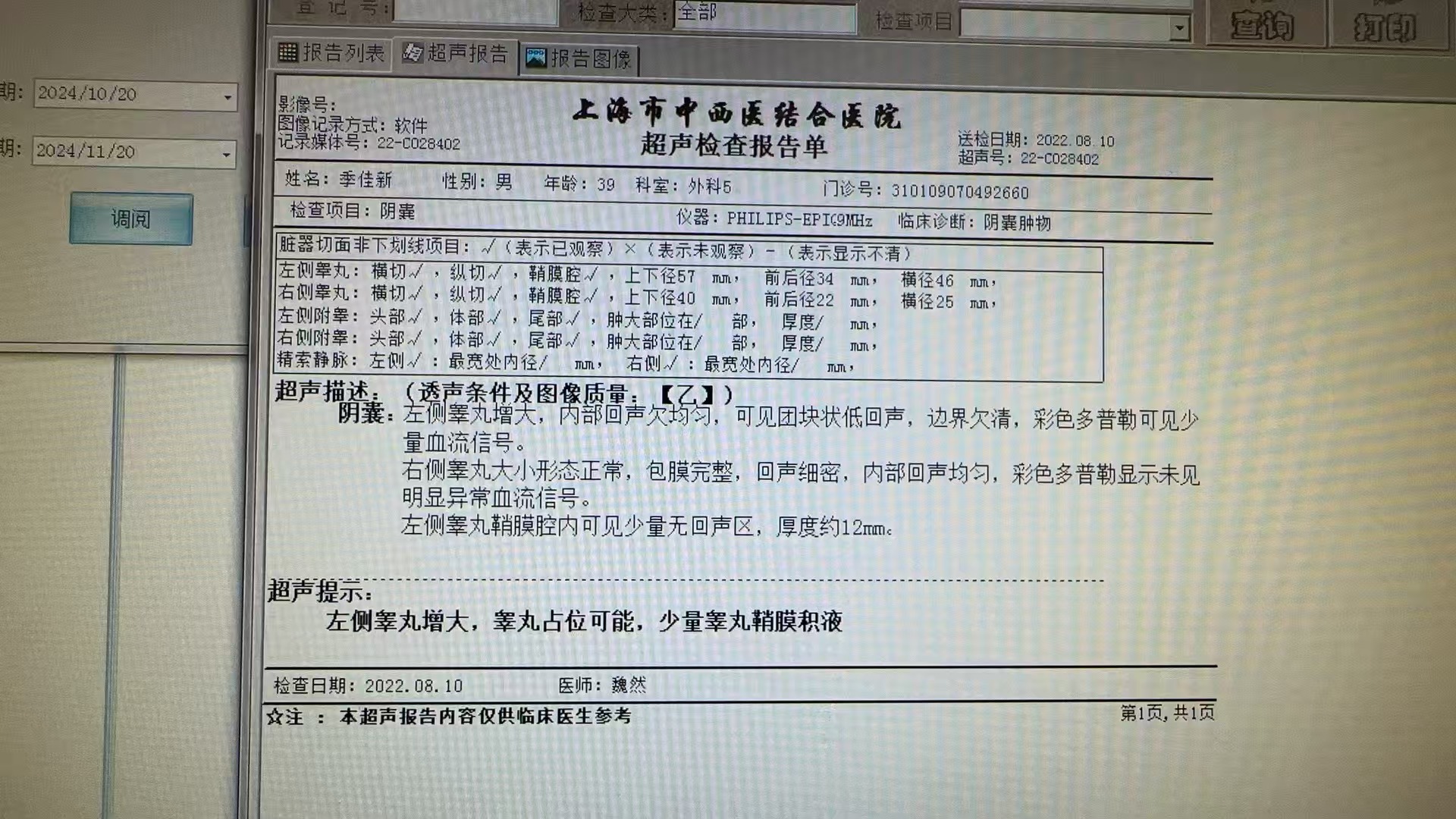


2022-8-10 Patient's Preoperative Ultrasound Report

Scrotum: The left testis is enlarged with uneven internal echoes, and a mass-like hypoechoic area is visible with unclear boundaries. Color Doppler shows a small amount of blood flow signal. The right testis is normal in size and shape, with an intact capsule and fine, uniform internal echoes. Color Doppler shows no significant abnormal blood flow signals. A small amount of anechoic area is visible in the left testicular sheath cavity, with a thickness of about 12mm.

Ultrasound Impression: Enlargement of the left testis, possible testicular mass, and a small amount of testicular sheath effusion.

MRI of cerebrum:


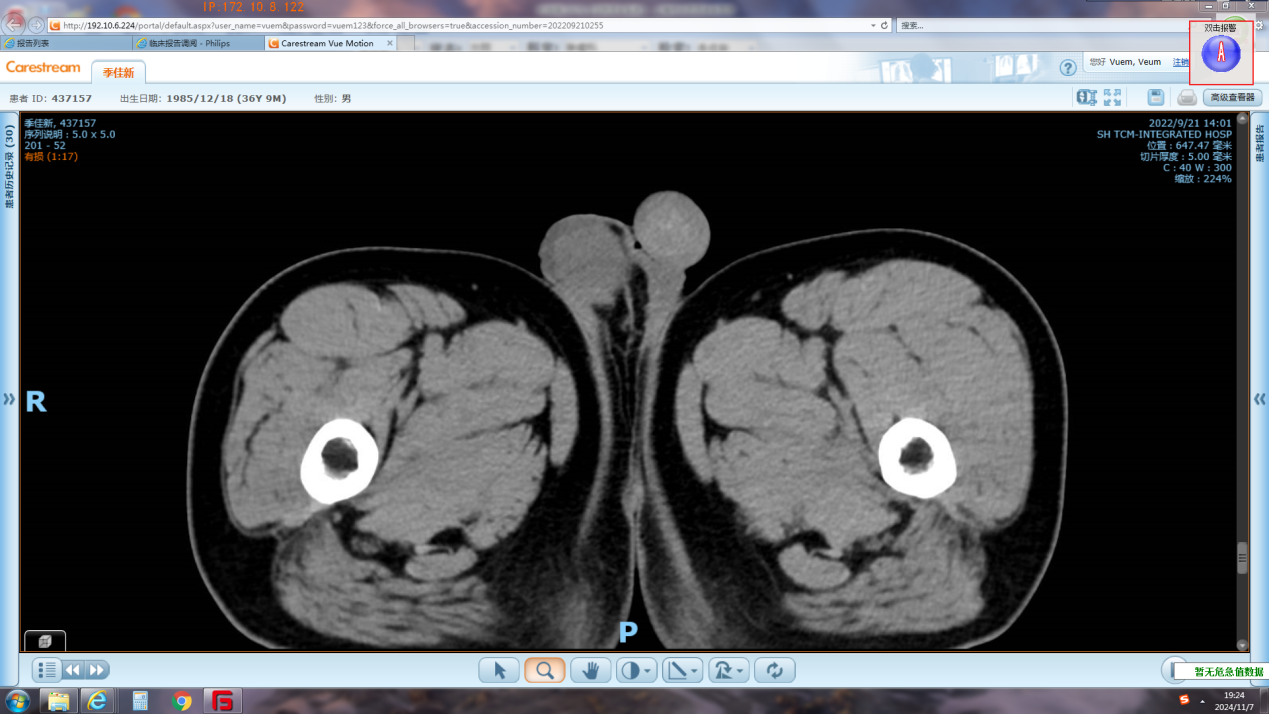


2022-09-21 Scrotum routine scan + enhancement: postoperative changes of left testicle


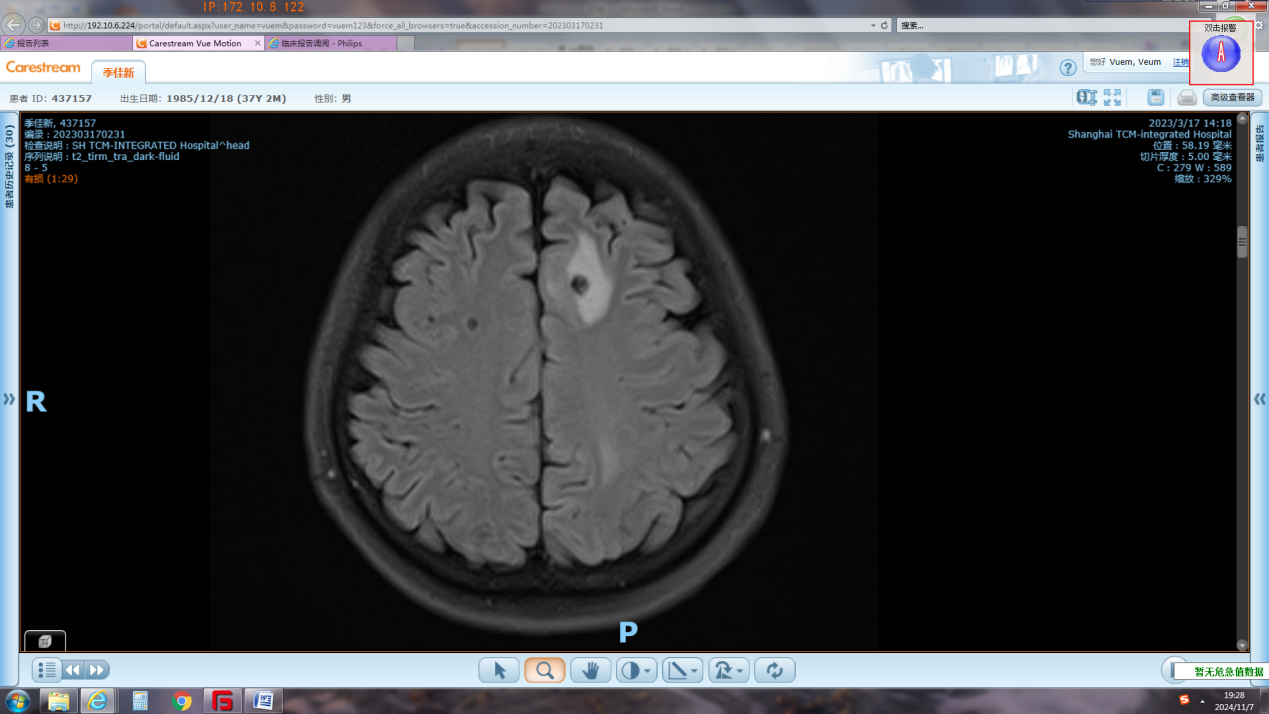


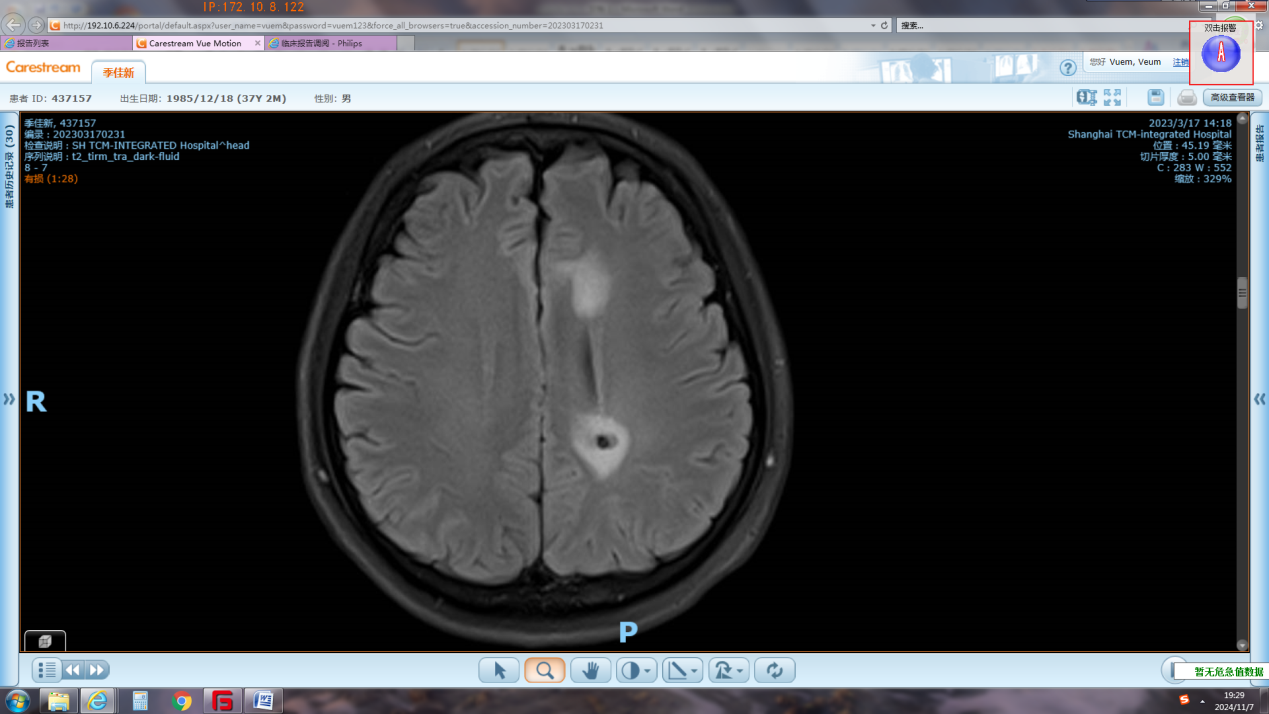


2023-03-17 Head MRI enhancement: multiple abnormal signals in the right cerebellar hemisphere and both cerebral hemispheres, considering metastasis combined with medical history.


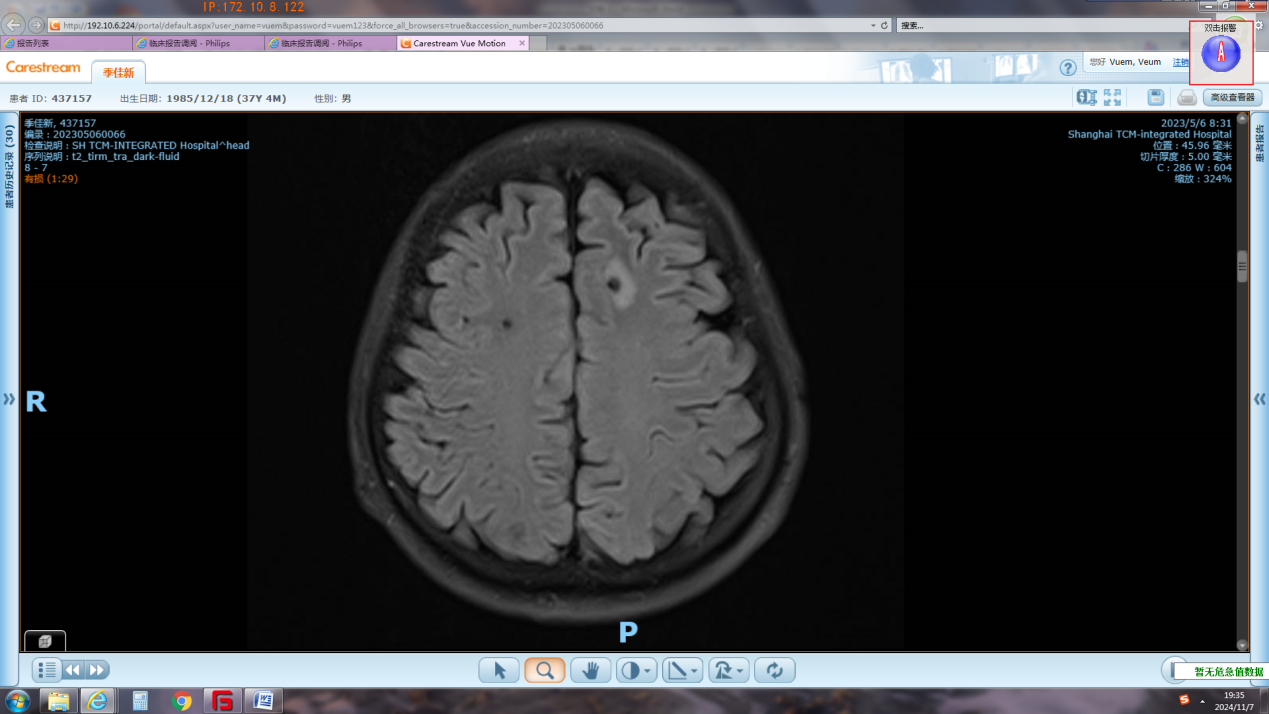


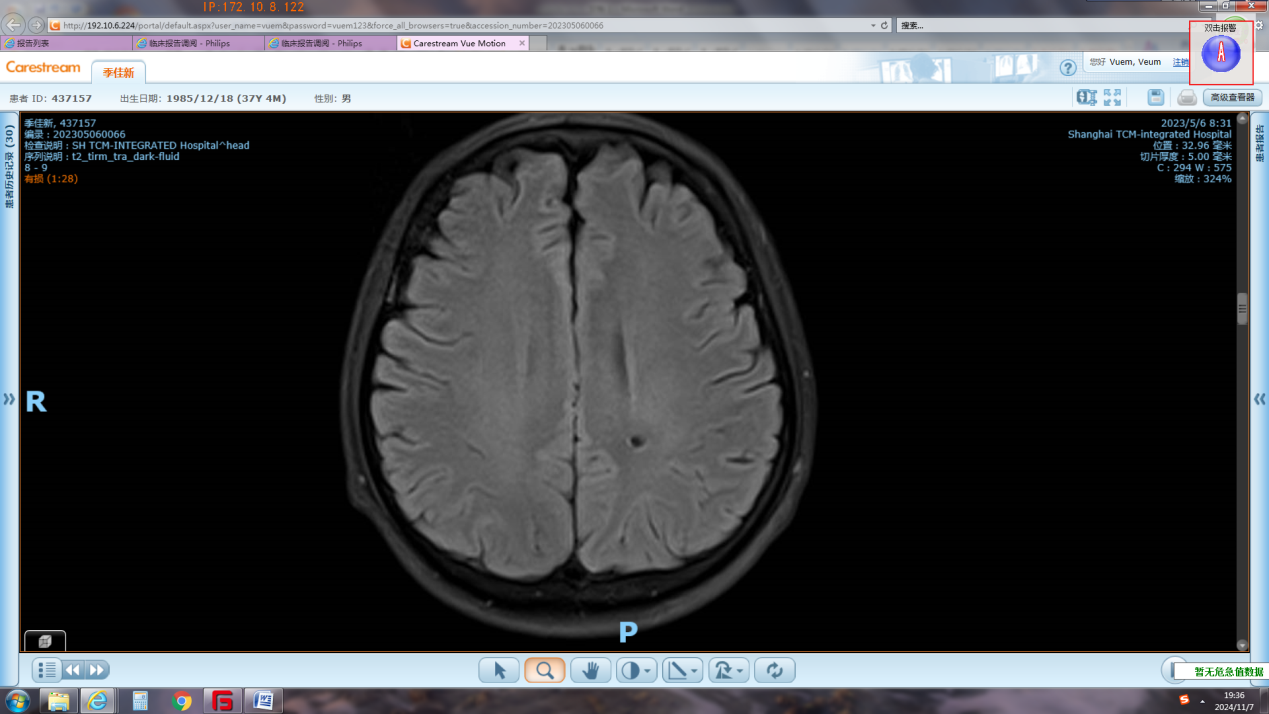


2023-05-06 Head MRI enhancement: multiple metastasies in the right cerebellar hemisphere and both cerebral hemispheres, improved with the previous film (2023-3-17), please combine clinical follow-up.
